# TNF-dependent hyperactivation of RIPK1-dependent cytotoxic signaling during embryogenesis and inflammation

**DOI:** 10.1371/journal.pbio.3001371

**Published:** 2021-08-31

**Authors:** Edward S. Mocarski, Pratyusha Mandal

**Affiliations:** Department of Microbiology and Immunology, Emory Vaccine Center, Emory University School of Medicine, Atlanta, Georgia, United States of America

## Abstract

In this issue of *PLOS Biology*, Zhang and colleagues unveil a complex midgestational death during embryogenesis of mice harboring caspase-8 cleavage-resistant receptor-interacting protein (RIP) kinase (RIPK)1. Tumor necrosis factor (TNF) receptor (TNFR)1-dependent signaling drives cell death through a novel pathway requiring synergism between apoptotic and pyroptotic caspases.

Over the past decade, receptor-interacting protein (RIP) kinase (RIPK)1 gained recognition as a key cell death signaling component contributing to developmental homeostasis and autoimmune disease, emerging as an attractive target for intervention with small molecule kinase inhibitors [1]. RIPK1, regulated by posttranslational modifications as well as cleavage by caspase-8, contributes to cell death signaling and inflammation via distinct protein–protein interaction domains in combination with enzymatic activity, as this perspective will summarize. In this current issue, Zhang and colleagues [2] investigate the implications of RIPK1 cleavage between the kinase and scaffold domains (D325 in mice and D324 in humans) [3]. Their story sheds light on a long-drawn saga with all the furtive features of an Agatha Christie novel, owing largely to the ability of RIPK1 to execute 3 mechanistically distinct outcomes downstream of tumor necrosis factor (TNF) receptor (TNFR)1: (i) caspase-8–dependent (extrinsic) apoptosis; (ii) RIPK3-dependent necroptosis; and (iii) activation of inflammatory cytokine production via nuclear factor kappa-light-chain-enhancer of activated B cells (NF-κB) and other transcription factors [1]. The new study sheds light on how RIPK1 toggles between these pathways, demonstrating that a cleavage-resistant mutant of RIPK1 (D325A; unable to be cleaved by caspase-8) forms a complex with RIPK3 and caspase-8 and triggers the activation of caspase-3 (a critical mediator of apoptosis) as well as caspase-1 and caspase-11 (critical mediators of pyroptosis). This causes developmental failure at around embryonic day 10. The results presented indicate that the cleavage of RIPK1 by caspase-8 restrains RIPK1-mediated activation of both apoptotic and pyroptotic caspases. Notably, collaboration between these same proteases contributes to endotoxic shock in mice where TNF and type I interferon act in combination to execute target cells within a few hours [4]. Furthermore, a single cleavage-resistant RIPK1 allele dramatically enhances susceptibility to inflammation in humans and mice, even though the second allele remains wild type [5–7]. While it remains to be resolved whether noncleavable RIPK1 mediates its gain-of-function effects because of increased stability or because of some novel structural feature conferring increased activity, the Zhang and colleagues study provides new insights into the mechanisms through which RIPK1 determines cell fate in the course of development and regulates inflammatory homeostasis throughout life.

RIPK1 contains an amino-terminal protein kinase domain and 2 distinct regions that mediate protein–protein interactions: a central receptor-interacting protein homotypic interaction motif (RHIM) and a carboxyl-terminal death domain (DD). RIPK1 kinase activity supports cell death signaling and cytotoxicity, acting in concert with the DD [1–3,5–7] and RHIM [8–10]. Each of these scaffold activities recruits additional adaptors that regulate cell death and inflammatory outcomes. In particular, RIPK1 RHIM recruits RIPK3 to unleash necroptosis while also restricting lethal consequences of Z-nucleic acid binding protein (ZBP)1 signaling [8–10]. The TNFR1-triggered signaling pathway depends on the engagement of receptor DD with the RIPK1 DD to initiate autophosphorylation and downstream signaling consequences (Fig 1). Although it is known that the elimination of RIPK1 kinase activity rescues TNF-induced pathology and cell death outcomes, as well as the embryonic lethality observed in mice expressing *Ripk1*^*D325A/D325A*^ homozygous mutation from embryonic day 10.5 (E10.5) to a couple of weeks after birth [5–7], RIPK1 kinase activity plays no role in the severe perinatal defects of *Ripk1*^*−/−*^ mice or in the phenotype of RIPK1 RHIM mutant mice [8–10]. Zhang and colleagues now show that the TNF-initiated E10.5 lethality of *Ripk1*^*D325A/D325A*^ mice is mediated by a RIPK1 scaffold- and kinase-dependent association with RIPK3, triggering apoptotic and pyroptotic caspase activation. Alternate RIPK3 kinase–dependent necroptosis is not involved at all. In addition, NF-κB signaling does not appear to contribute to the embryonic lethality or aberrant signaling conferred by *Ripk1*^*D325A/D325A*^, even though this transcription factor has long been viewed as dependent on RIPK1 function in the TNF pathway [1–3,5–7]. Thus, noncleavable RIPK1 retains the ability to sustain NF-κB–initiated prosurvival signaling. It is noteworthy that mice with mutated RIPK1 RHIM, like *Ripk1*^*D325A/D325A*^ mice, manifest a RIPK3-dependent dysregulation of embryogenesis. Unexpectedly, dysregulated development in RIPK1 RHIM mutant mice is driven by the RHIM-containing sensor protein ZBP1, even though ZBP1 is itself completely dispensable for mammalian life [8]. Whether ZBP1 contributes to the developmental defects observed in *Ripk1*^*D325A/D325A*^ mice remains to be seen. Nonetheless, a remarkable picture emerges from these studies depicting how much control RIPK1, by regulating RIPK3, exerts over TNF-dependent, caspase-8–mediated signaling outcomes.

**Fig 1:**
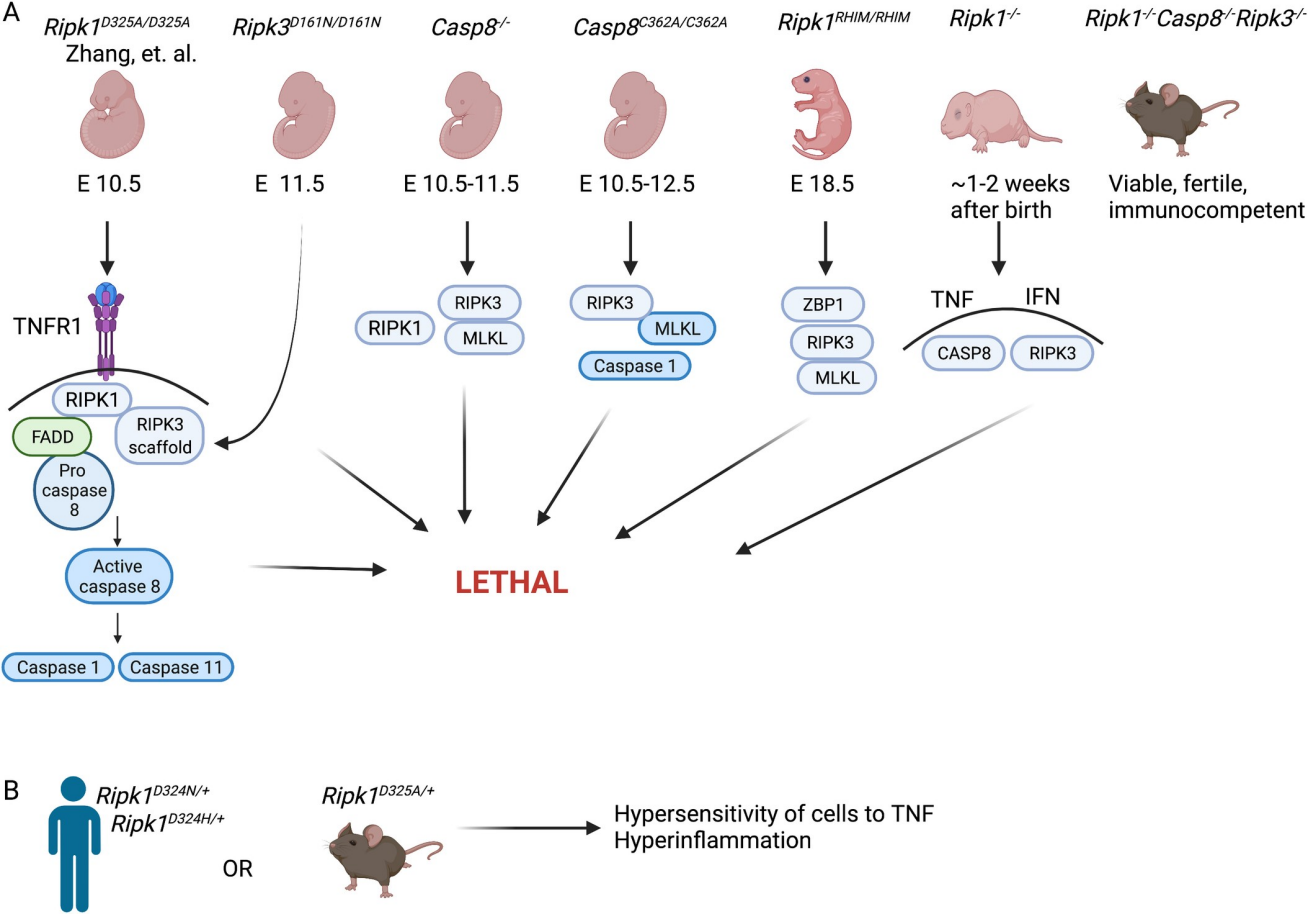
RIPK1 dictates cell fate and inflammatory outcome by restricting cell death components. **(A)** Summary of consequences with indicated mutations introduced in RIPK1 as described by Zhang and colleagues and others [1–3,5–9], RIPK3 [10], and CASP8 [5–7] genes. Genetic requirements for execution of lethality are indicated for each condition. **(B)** Outcome of heterozygous cleavage-resistant RIPK1 alleles [5–7]. Original images created with the assistance of BioRender.com. CASP8, caspase-8; E10.5, embryonic day 10.5; E11.5, embryonic day 11.5; E12.5, embryonic day 12.5; E18.5, embryonic day 18.5; FADD, Fas-associated death domain protein; IFN, interferon; MLKL, mixed lineage kinase domain-like; RIP, receptor-interacting protein; RIPK, receptor-interacting protein kinase; TNF, tumor necrosis factor; TNFR, tumor necrosis factor receptor; ZBP, Z-nucleic acid binding protein.

The recent era of our understanding of how programmed cell death pathways control mammalian development began when midgestational defects during embryogenesis of caspase-8–deficient mice were fully reversed by eliminating expression of RIPK3, and the parturition defects of RIPK1-deficient mice were rescued by combined elimination of both caspase-8 and RIPK3 [9]. Notably, the lethal consequences resulting from RIPK1 deficiency depend on combined TNF and type I interferon signaling. Despite the absence of RIPK1 and 2 other critical extrinsic cell death signaling components (RIPK3 and caspase-8), *Ripk1*^*−/−*^*Casp8*^*−/−*^*Ripk3*^*−/−*^ mice show a remarkable level of immunocompetence, retaining a full complement of hematopoietic cell types and mounting an aggressive adaptive immune response that leads to control over murine cytomegalovirus [9]. Thus, RIPK1 cannot be viewed as a vital protein essential for mammalian life, but rather as a rheostat or checkpoint preventing hypersensitivity to inflammatory signals throughout development and life (Fig 1). In this light, cleavage-resistant RIPK1 nucleates an aberrant signaling platform that either fails to check RIPK3 and caspase-8 or that promotes the hyperactivity of these signaling effectors. The hyperinflammation observed in patients carrying an allele of cleavage-resistant RIPK1 results in inflammatory amplification via interleukin-6 receptor-dependent pathways [5–7]. The noted collaboration between interferons and TNF during endotoxemia or parturition [9,10] suggest that a similar synergy of cytokines may underlie RIPK1-dependent signaling involved in mammalian development. Although the extent of crosstalk between inflammatory cytokines remains to be determined, the current work identifies the RIPK1-regulated network in additional nodes beyond what was known, warranting continued investigation. Illuminating these nodes stands to provide new opportunities for therapeutic intervention in TNF-mediated pathologies and other chronic inflammatory conditions.

## Abbreviations

DD: death domain
E10.5: embryonic day 10.5
NF-κB: nuclear factor kappa-light-chain-enhancer of activated B cells
RHIM: receptor-interacting protein homotypic interaction motif
RIP: receptor-interacting protein
RIPK: receptor-interacting protein kinase
TNF: tumor necrosis factor
TNFR: tumor necrosis factor receptor
ZBP: Z-nucleic acid binding protein
